# Use of patient-reported outcome measures after breast reconstruction in low- and middle-income countries: a scoping review

**DOI:** 10.1186/s41687-024-00687-y

**Published:** 2024-02-28

**Authors:** Sri Harshini Malapati, Colby J. Hyland, George Liang, Maria O. Edelen, Amanda Fazzalari, Manraj N. Kaur, Paul A. Bain, Gita N. Mody, Andrea L. Pusic

**Affiliations:** 1 Brigham and Women’s Hospital, Harvard Medical School, 75 Francis St., Boston, MA 02115 USA; 2 Countway Library, Harvard Medical School, Boston, MA USA; 3 Department of Surgery, Division of Cardiothoracic Surgery, University of North Carolina at Chapel Hill, Chapel Hill, NC USA

## Abstract

**Background:**

Patient-reported outcome measures (PROMs) are increasingly administered in high-income countries to monitor health-related quality of life of breast cancer patients undergoing breast reconstruction. Although low- and middle-income countries (LMICs) face a disproportionate burden of breast cancer, little is known about the use of PROMs in LMICs. This scoping review aims to examine the use of PROMs after post-mastectomy breast reconstruction among patients with breast cancer in LMICs.

**Methods:**

MEDLINE, Embase, Web of Science, CINAHL, and PsycINFO were searched in August 2022 for English-language studies using PROMs after breast reconstruction among patients with breast cancer in LMICs. Study screening and data extraction were completed. Data were analyzed descriptively.

**Results:**

The search produced 1024 unique studies, 33 of which met inclusion criteria. Most were observational (48.5%) or retrospective (33.3%) studies. Studies were conducted in only 10 LMICs, with 60.5% in China and Brazil and none in low-income countries. Most were conducted in urban settings (84.8%) and outpatient clinics (57.6%), with 63.6% incorporating breast-specific PROMs and 33.3% including breast reconstruction-specific PROMs. Less than half (45.5%) used PROMs explicitly validated for their populations of interest. Only 21.2% reported PROM response rates, ranging from 43.1 to 96.9%. Barriers and facilitators of PROM use were infrequently noted.

**Conclusions:**

Despite the importance of PROM collection and use in providing patient-centered care, it continues to be limited in middle-income countries and is not evident in low-income countries after breast reconstruction. Further research is necessary to determine effective methods to address the challenges of PROM use in LMICs.

**Supplementary Information:**

The online version contains supplementary material available at 10.1186/s41687-024-00687-y.

## Background

Breast cancer is the leading cause of cancer among women worldwide, with a disproportionate impact in low- and middle-income countries (LMICs) [1]. Timely diagnosis of breast cancer is often limited in LMICs due to health system and sociocultural barriers, including healthcare costs, lack of access to hospitals, referral delays, and concerns of discrimination related to cancer diagnosis [2–8]. Many patients with breast cancer diagnoses undergo mastectomy, which can adversely affect well-being including body image and sexual health [9]. To improve overall health-related quality of life (HRQL) among these patients, breast reconstruction can be performed. Given that HRQL is best assessed by patients, changes in HRQL after breast reconstruction can then be monitored by measuring patient-reported outcomes (PROs).

PROs are reports of patient health status that are directly provided by patients without interpretation by anyone else [10]. PROs are captured by utilizing validated questionnaires known as patient-reported outcome measures (PROMs), which measure health outcomes including physical and psychosocial wellbeing [10]. PROMs are being increasingly utilized in routine clinical care in high-income countries (HICs), as they have been shown to promote patient engagement, experience, and shared decision-making [11–13]. PROMs are particularly relevant in the context of surgery, given that surgical interventions can impact multiple aspects of health status within a short period of time. The administration of PROMs is especially important in breast surgery as with overall improvements in survival rates and adverse events, measurement of the quality of surgical care has been shifting from morbidity and mortality rates to patient-reported outcomes including HRQL [14].

Given that breast reconstruction primarily aims to improve HRQL, the use of PROMs in conjunction with routine breast reconstruction is critical to comprehensively understand patient outcomes and inform quality improvement. PROMs have gained considerable traction in the HICs as a means to measure the impact of breast reconstruction on PROs. As such, PROMs have provided valuable insights on the selection of autologous versus implant-based reconstruction, saline versus silicone implants, fat grating, and patient education [15]. However, although LMICs face disproportionately high incidence, morbidity, and mortality of breast cancer [16], there is limited understanding of the use of PROMs among patients with breast cancer in LMICs. As such, improving surgical equity and patient outcomes globally will depend, in part, on understanding PROM usage in LMICs. This study, therefore, aims to review the literature to examine the current utilization of PROMs related to breast reconstruction among patients with breast cancer in LMICs. More specifically, this study aims to characterize the patient populations and PROMs included in the studies, as well as the geographical locations at which PROMs are used. This review will improve our present understanding of PROM use and elucidate potential areas of improvement to facilitate PROM use in LMICs.

## Methods

This scoping review was performed according to the Joanna Briggs Institute methodology and reported in compliance with the Preferred Reporting Items for Systematic Reviews and Meta-Analyses for Scoping Review (PRISMA-ScR) checklist [17, 18].

### Search strategy

Studies reporting on the use of PROMs for breast reconstruction in LMICs were identified by searching the electronic databases MEDLINE (Ovid), Embase (Elsevier), Web of Science Core Collection (Clarivate), CINAHL Complete (EBSCO), and PsycINFO (EBSCO). The searches included terms for PROMs and breast reconstruction for breast cancer, limited to studies in LMICs as defined and categorized by the World Bank [19] (Supplementary Table 1). Relevant controlled vocabulary terms were included when available; no date limits were applied. The search was last run on August 28, 2022.

### Study selection

All studies identified using the search strategy were imported into the systematic review management tool, Covidence (Veritas Health Innovation, Melbourne, Australia). Inclusion and exclusion criteria were predefined. Accordingly, titles and abstracts were screened by two independent reviewers (SM, GL), and conflicts were resolved by a third independent reviewer (CJH). Subsequently, two independent reviewers (SM, GL) reviewed the full texts, and conflicts were resolved by discussion among reviewers.

### Study eligibility

Inclusion criteria for studies were: (1) published in English, (2) conducted in LMICs as defined by the World Bank in 2022, and (3) reported the use of PROMs to measure outcomes related to breast reconstruction among patients with breast cancer. Exclusion criteria included (1) studies with only one question, rather than multiple items, related to PROs, (2) articles focused on breast reconstruction among patients without history of breast cancer, and (3) non-primary literature, theses, dissertations, conference abstracts, and editorials.

### Data analyses

Study variables of interest were determined prior to data extraction. For each study, the following were collected if available: study authors, publication year, journal, study aims, patient characteristics, study location, PROM characteristics, facilitators and barriers of PROM use, and cultural relevance of the utilized PROM. Descriptive analyses were performed. The American Society of Plastic Surgeons (ASPS) Evidence Rating Scales [20] were used to identify the level of evidence for each study.

## Results

### Search results

**Fig. 1:**
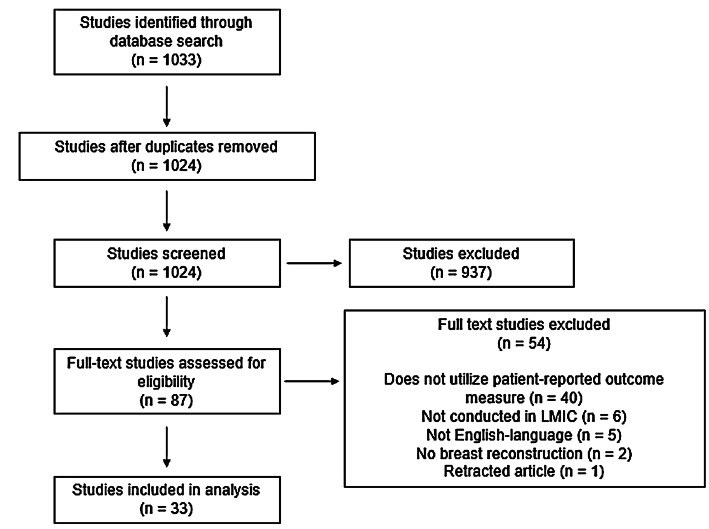
PRISMA diagram for included studies. *PRISMA* Preferred Reporting Items for Systematic Reviews and Meta-Analyses

The search resulted in 1024 unique studies (Fig. 1). Full-text review was conducted for 83 articles, yielding 33 studies that were included in this study.

### Study characteristics

The characteristics of included studies are shown in Table 1. Studies were published between 2001 and 2022. Most studies were cross-sectional observational studies with level 3 evidence (n = 16, 48.5%), followed by retrospective studies with level 3 evidence (n = 11, 33.3%) and prospective cohort studies with level 2 evidence (n = 6, 18.2%). Studies included sample sizes ranging from four to 469. The mean/median age of included populations ranged from 30 to 58 years. Most studies did not specify the educational attainment of the included population (n = 22, 66.7%). There were two studies (6.0%) in which the majority of included patients had educational attainment lower than high school.

**Table 1:** Characteristics of included studies (n = 33)

Author, year	Study aims	Sample size	Mean/median age	Majority with high school education	Study setting	Study type	Level of evidence
AbuElnga 2021 [21]	Assess oncologic safety and cosmetic outcomes of extreme oncoplastic therapeutic mammoplasty among patients initially scheduled for mastectomy	36	48.5	Unspecified	Urban	Prospective cohort study	2
Aguiar 2017 [22]	Assess patient-reported outcomes measured by BREAST-Q after implant-based breast reconstruction	114	43	Yes	Urban	Cross-sectional study	3
Archangelo 2019 [23]	Evaluate sexual function, body image, and depression after postmastectomy breast reconstruction	90	48	Unspecified	Urban	Cross-sectional study	3
Athamnah 2021 [24]	Determine clinical outcome, patient satisfaction, and cancer recurrence after nipple-sparing mastectomy	4	30	Unspecified	Urban	Prospective cohort study	2
Chang 2007 [25]	Examine health-related quality of life and attitudes toward breast surgery among breast cancer patients	235	49	Unspecified	Mixed	Cross-sectional study	3
Cortes-Flores 2014 [26]	Determine quality of life among patients treated with one of three different types of surgery for breast cancer	139	48.7	Unspecified	Unspecified	Cross-sectional study	3
Cortes-Flores 2017 [27]	Evaluate and compare the sexuality of women who underwent conservative mastectomy, mastectomy alone, and those who had breast reconstruction after cancer treatment	74	45	Unspecified	Unspecified	Cross-sectional study	3
Denewer 2012 [28]	Evaluate whether immediate autologous breast reconstruction influences QOL and patient satisfaction outcomes among women with breast cancer in comparison to the traditional mastectomy	200	39 (group 1, w/ recon), 51.5 (group 2, no recon)	Unspecified	Urban	Prospective cohort study	2
Fontes 2019 [29]	Assess the influence of different surgical treatment modalities on the level of physical activity, functional capacity, and quality of life of breast cancer survivors	180	Median BR group: 52	Yes	Urban	Cross-sectional study	3
Fung 2001 [30]	Investigate the effects that different types of breast surgery have on the quality of life of Chinese women	49	44.2	Unspecified	Urban	Retrospective cohort study	3
Hashem 2017 [31]	Compare cosmetic outcomes and patient satisfaction between batwing mammoplasty and Wise pattern mammoplasty	126	44 (Wise pattern mammoplasty), 47 (Batwing mammoplasty)	Unspecified	Urban	Retrospective cohort study	3
He 2017 [32]	Identify associations between radiation, surgery timing relative to radiation, and autologous breast reconstruction	360	42	Unspecified	Urban	Retrospective cohort study	3
He 2019 [33]	Investigate the aesthetic outcomes within implant-based breast reconstruction patients who underwent a novel selection method	135	38.9 ± 8.3 years	Unspecified	Unspecified	Retrospective cohort study	3
He 2021 [34]	Investigate the oncological safety of immediate breast reconstruction, and to compare the survival and surgical outcomes between implant-based and autologous reconstruction	124	38.4 (implant group), 41.7 (autologous group)	Unspecified	Urban	Retrospective cohort study	3
Koppiker 2019 [35]	Investigate post-surgery complications after 1 year in patients who have undergone IBRS-ALDS (autologous lower dermal sling) and RT (radiation therapy)	78	Group A mean = 49.5, Group B mean = 47.2	Unspecified	Urban	Retrospective cohort study	3
Kovacevic 2020 [36]	Determine the relationship between the levels of perceived quality of life in patients operated on for breast cancer in relation to the type of surgery, using the standardized questionnaires	425	58	Yes	Urban	Retrospective cohort study	3
Li 2021 [37]	Evaluate the feasibility, complications, and cosmetic outcomes of immediate autologous fat grafting during breast-conserving surgery (BCS) in Chinese patients with early-stage breast cancer	58	45	Unspecified	Urban	Retrospective cohort study	3
Liu 2021 [38]	Examine the feasibility of mastoscopic modified radical mastectomy (MRM) with skin nipple-areola preservation under air cavity-free suspension hook and stage I silicone prosthesis implantation (SMALND) compared with routine MRM	87	43.7	Unspecified	Urban	Retrospective cohort study	3
Macedo 2018 [39]	Evaluate sexual dysfunction among breast cancer patients with mastectomy, with or without breast reconstruction	28	53.77	Unspecified	Urban	Cross-sectional study	3
Manganiello 2011 [40]	Evaluate the sexual functioning of breast cancer patients post mastectomy and its association with their quality of life	100	Unspecified	Unspecified	Urban	Cross-sectional study	3
Medina-Franco 2010 [41]	Compare patient-reported body image and quality of life by breast surgery type	202	54	No	Urban	Cross-sectional study	3
Noyan 2006 [42]	Assess patient satisfaction by breast surgery type	125	41	No	Urban	Cross-sectional study	3
Ortega 2018 [43]	Assess work ability and productivity after breast surgery among breast cancer patients	152	47.5–50.1, based on group	Yes	Urban	Cross-sectional study	3
Ou 2015 [44]	Examine oncological and cosmetic outcomes in Asian women who underwent nipple-sparing mastectomy in Taiwan	42	45.2	Unspecified	Urban	Retrospective cohort study	3
Ozturk 2016 [45]	Identify differences in sexual function between postmastectomy breast reconstruction and breast-conserving surgery or mastectomy alone	100	47	Unspecified	Urban	Cross-sectional study	3
Paulinelli 2021 [46]	Evaluate the results of a cohort of patients submitted to a new technique of oncoplastic mammoplasty, referred to as Disguised Geometric Compensation Mammoplasty	25	47	Unspecified	Urban	Prospective cohort study	2
Shi 2011 [47]	Examine changes in long-term patient responses and predictors of quality of life outcomes after breast surgery	132	47.70 to 53.84, based on group	Unspecified	Unspecified	Prospective cohort study	2
Sinaei 2017 [48]	Examine quality of life among breast cancer patients with breast reconstruction	146	48.21	Yes	Urban	Cross-sectional study	3
Srimontayamas 2017 [49]	Examine long-term effects of different surgical treatments on QoL in Thai women with breast cancer	265	Mastectomy group: 49, BCT group: 47, Mastectomy-TRAM: 44	Yes	Urban	Cross-sectional study	3
Wang 2022 [50]	Determine incidence of chronic post-surgical pain after single-stage implant-based breast reconstruction	159	40.94	Unspecified	Urban	Retrospective cohort study	3
Yang 2015 [51]	Measure patient satisfaction by breast reconstruction type	285	Unspecified	Yes	Urban	Cross-sectional study	3
Zhang 2015 [52]	Evaluate associations between psychological functioning and patient satisfaction with breast reconstruction	264	44.7	Yes	Urban	Prospective cohort study	2
Zhuang 2022 [53]	Assess decisional conflict, decision regret, self-stigma, and quality of life by breast surgery type	469	46.15	Yes	Urban	Cross-sectional study	3

**Fig. 2:**
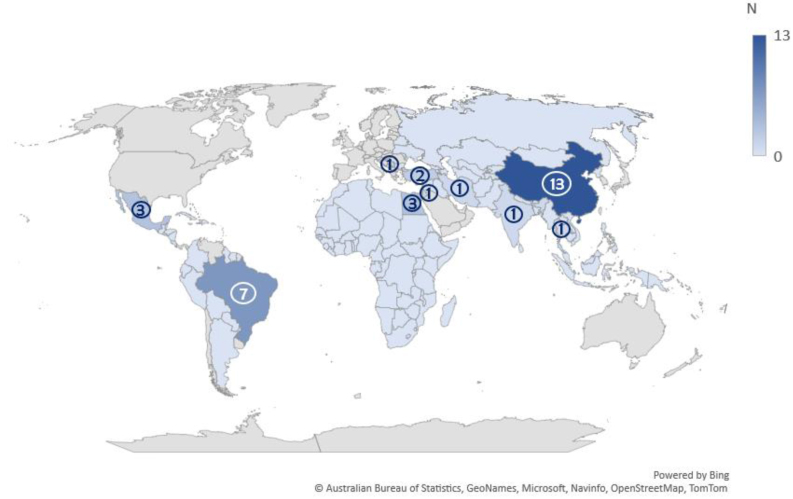
Distribution of studies, by country. *Of note, 3 of the studies in China were conducted in Taiwan. Although Taiwan is technically considered China on a national level, the resources and income level of Taiwan may differ greatly from mainland China

Studies represented five continents (North America, South America, Europe, Africa, and Asia). Most studies were conducted in China (n = 13, 39.3%), followed by Brazil (n = 7, 21.2%). Three studies each were conducted in Egypt (9.0%) and Mexico (9.0%), two studies in Turkey (6.0%), and one study each (3.0%) in India, Iran, Jordan, Serbia, and Thailand (Fig. 2). Most studies were conducted in urban settings (n = 28, 84.8%), as defined by the World Bank as areas with a minimum population of 50,000 residents in continuous grid cells—over 1500 residents for every km^2^ [54] (Table 1).

### PROM characteristics

The characteristics of the utilized PROMs are included in Table 2. We identified 35 unique PROMs across the studies, with 16 (48.5%) studies using multiple PROMs. The most frequently used PROM was the BREAST-Q (n = 8, 24.2%), followed by the Female Sexual Function Index (FSFI) (n = 4, 16.7%) and the Functional Assessment of Cancer Therapy-Breast (FACT-B) (n = 4, 16.7%). Of the 33 total studies, 21 (63.6%) incorporated a breast-specific PROM, with 11 (33.3%) administering a breast reconstruction-specific PROM. While most of the studies utilized a validated PROM (n = 30, 90.5%), only 15 (45.5%) studies used a PROM that was explicitly validated for their population of interest (e.g., country or language).

**Table 2:** Characteristics of utilized PROM(s)

Author, year	PROM(s) used	Breast-specific PROM used?	Breast-reconstruction-specific PROM used?	Used validated PROM(s)	Used PROM(s) validated for country/language of interest
AbuElnga 2021 [21]	Custom questionnaire	✓	✓		
Aguiar 2017 [22]	BREAST-Q	✓	✓	✓	✓
Archangelo 2019 [23]	Female Sexual Function Index (FSFI), Beck Depression Inventory (BDI), Body Dysmorphic Disorder Examination (BDDE)			✓	✓
Athamnah 2021 [24]	BREAST-Q	✓	✓	✓	Unspecified
Chang 2007 [25]	Functional Assessment of Chronic Illness Therapy–Breast, traditional Chinese version 4 (FACIT-B)	✓		✓	Unspecified
Cortes-Flores 2014 [26]	European Organization for Research and Treatment of Cancer Quality of Life Questionnaire, core version 30 (EORTC QLQ-C30), EORTC QLQ-BR23	✓		✓	✓
Cortes-Flores 2017 [27]	FSFI (Female Sexual Function Index)			✓	✓
Denewer 2012 [28]	Breast impact of treatment scale (BITS), Body satisfaction scale (BSS)	✓		✓	
Fontes 2019 [29]	International Physical Activity Questionnaire (IPAQ), Health Assessment Questionnaire (HAQ-20), Medical Outcomes Study 36-item Short Form Health Survey (SF-36)			✓	✓
Fung 2001 [30]	Chinese health questionnaire (CHQ-12) for psychological well-being			✓	✓
Hashem 2017 [31]	Custom questionnaire	✓	✓		
He 2017 [32]	BREAST-Q	✓	✓	✓	Unspecified
He 2019 [33]	BREAST-Q	✓	✓	✓	Unspecified
He 2021 [34]	BREAST-Q	✓	✓	✓	Unspecified
Koppiker 2019 [35]	BREAST-Q	✓	✓	✓	Unspecified
Kovacevic 2020 [36]	The World Health Organization Quality of Life-Bref (WHOQOL-bref), Functional Assessment of Cancer Therapy-Breast (FACT-B)	✓		✓	Unspecified
Li 2021 [37]	BREAST-Q	✓	✓	✓	Unspecified
Liu 2021 [38]	Functional Assessment of Cancer Therapy-Breast (FACT-B)	✓		✓	✓
Macedo 2018 [39]	Adapted Etienne and Waitman (2006) assessment card and Female Sexual Function Index (FSFI)			✓	✓
Manganiello 2011 [40]	Sexual Quotient Female Version (SQ-F), Medical Outcomes Study 36-item Short Form Health Survey (SF-36)			✓	✓
Medina-Franco 2010 [41]	Body image scale and 36-items Short Form Health Survey (SF-36)			✓	Unspecified
Noyan 2006 [42]	Structured Clinical Interview for DSM-IV, Clinical Version (SCID-I), the Body Cathexis Scale (BCS); and the Rosenberg Self-Esteem Scale (RSE)			✓	Unspecified
Ortega 2018 [43]	WPAI-GH questionnaire, WLQ			✓	✓
Ou 2015 [44]	Custom questionnaire				
Ozturk 2016 [45]	Female Sexual Function Index questionnaire (FSFI)			✓	✓
Paulinelli 2021 [46]	BREAST-Q	✓	✓	✓	✓
Shi 2011 [47]	European Organization for Research and Treatment of Cancer Quality of Life Questionnaire, core version 30 (EORTC QLQ-C30), EORTC QLQ-BR23	✓		✓	✓
Sinaei 2017 [48]	European Organization for Research and Treatment of Cancer Quality of Life Questionnaire, core version 30 (EORTC QLQ-C30), EORTC QLQ-BR23	✓		✓	✓
Srimontayamas 2017 [49]	Functional Assessment of Cancer Therapy-Breast (FACT-B), Functional Assessment of Cancer Therapy - Genera (FACT-G)	✓		✓	Unspecified
Wang 2022 [50]	BREAST-QTM-BREAST CANCER CORE SCALE VERSION 2.0	✓		✓	Unspecified
Yang 2015 [51]	Michigan Breast Reconstruction Outcomes Study tool (MBROS)	✓	✓	✓	Unspecified
Zhang 2015 [52]	Rosenberg Self-Esteem Scale, 3-item subset of the Hopwood Body Image Scale, Patient Health Questionnaire nine-item (PHQ-9), Generalized Anxiety Disorder seven-item (GAD-7), Alderman scale			✓	Unspecified
Zhuang 2022 [53]	Decisional Conflict Scale; Decision Regret Scale, Self-Stigma Form, Functional Assessment of Cancer Treatment-B (FACT-B)	✓		✓	✓

*PROM* patient-reported outcome measures

### PROM administration

Details regarding PROM administration are listed in Table 3. PROMs were most often administered in an outpatient clinic setting (n = 19, 57.6%). Other studies involved the completion of PROMs remotely (n = 11, 33.3%), with the administration via telephone (n = 4, 12.1%), mail (n = 3, 9.1%), or online platform (n = 2, 6.1%). PROMs were either self-administered (n = 11, 33.3%) or administered via interview by a clinician or a member of the research team (n = 13, 39.4%). Seven studies (21.2%) measured PROM response rates, which ranged from 43.1 to 96.9%. Two studies (6.1%) included the percentage of patients lost to follow-up, which ranged from 2.5 to 90.5%.

**Table 3:** Characteristics of PROM administration

Author, year	Method of administration	Setting of administration	Completion by self or proxy	Remote vs. in-person completion	Completion rate	% Lost to follow-up
AbuElnga 2021 [21]	Unspecified	Outpatient clinic	Unspecified	In-person	N/A	N/A
Aguiar 2017 [22]	Unspecified	Unspecified	Self	In-person	N/A	N/A
Archangelo 2019 [23]	Interview	Outpatient clinic	Proxy	In-person	N/A	N/A
Athamnah 2021 [24]	Unspecified	Outpatient clinic	Self	In-person	N/A	N/A
Chang 2007 [25]	Unspecified	Outpatient clinic	Self	In-person	93.6%	N/A
Cortes-Flores 2014 [26]	Unspecified	Outpatient clinic or at home	Self	In-person	N/A	N/A
Cortes-Flores 2017 [27]	Unspecified	Outpatient clinic	Self	In-person	N/A	N/A
Denewer 2012 [28]	Unspecified	Outpatient clinic	Proxy	In-person	N/A	N/A
Fontes 2019 [29]	Unspecified	Unspecified	Unspecified	Unspecified	N/A	N/A
Fung 2001 [30]	Interview (in-person or phone call)	Outpatient clinic or at home	Proxy	In-person or remote	64%	N/A
Hashem 2017 [31]	Unspecified	Unspecified	Unspecified	Unspecified	N/A	N/A
He 2017 [32]	Unspecified	Outpatient clinic	Unspecified	In-person	N/A	N/A
He 2019 [33]	Unspecified	Unspecified	Unspecified	In-person	N/A	N/A
He 2021 [34]	Unspecified	Outpatient clinic or at home	Unspecified	In-person	N/A	N/A
Koppiker 2019 [35]	Unspecified	Unspecified	Unspecified	Unspecified	N/A	N/A
Kovacevic 2020 [36]	Interview	Outpatient clinic	Proxy	In-person	N/A	N/A
Li 2021 [37]	Unspecified	Unspecified	Unspecified	Unspecified	N/A	N/A
Liu 2021 [38]	Unspecified	Unspecified	Self	Unspecified	N/A	N/A
Macedo 2018 [39]	Unspecified	Waiting room	Self	In-person	N/A	N/A
Manganiello 2011 [40]	Unspecified	Outpatient clinic or at home	Unspecified	In-person	N/A	N/A
Medina-Franco 2010 [41]	Interview	Unspecified	Proxy	Unspecified	N/A	N/A
Noyan 2006 [42]	Interview	Outpatient clinic	Proxy	In-person	N/A	N/A
Ortega 2018 [43]	Unspecified	Outpatient clinic	Self	In-person	N/A	N/A
Ou 2015 [44]	Mail and telephone	Home	Self or proxy	Remote	N/A	N/A
Ozturk 2016 [45]	Interview	Home	Proxy	Remote	N/A	N/A
Paulinelli 2021 [46]	Unspecified	Unspecified	Unspecified	Remote	64%	N/A
Shi 2011 [47]	Unspecified	Unspecified	Proxy	Unspecified	N/A	22.5%
Sinaei 2017 [48]	Interview	Home	Proxy	Remote	43.1%	N/A
Srimontayamas 2017 [49]	Interview	Outpatient clinic	Proxy	In-person	N/A	N/A
Wang 2022 [50]	Online or telephone	Home	Self	Remote	87.4%	N/A
Yang 2015 [51]	Letter and telephone	Outpatient clinic or at home	Proxy	In-person or remote	72.6%	N/A
Zhang 2015 [52]	Unspecified	Outpatient clinic or at home	Proxy	In-person or remote	N/A	90.5%
Zhuang 2022 [53]	Paper and online	Outpatient clinic or at home	Self	In-person or remote	96.9%	N/A

*PROM* patient-reported outcome measure; *N/A* not available

## Discussion

The current scoping review evaluated the studies that have utilized PROMs among breast cancer patients with breast reconstruction in LMICs. Notably, our study found that the use of PROMs for breast reconstruction in LMICs has only been reported in 10 LMICs, with 60.5% studies conducted in China and Brazil, and 84.8% studies conducted in urban settings. Moreover, although 90.5% of studies used a validated PROM, only 45.5% used a PROM that was explicitly validated for the country and/or language of administration. PROM response rates as well as barriers and facilitators of PROM use were infrequently mentioned. Our findings highlight that the use of PROMs after breast reconstruction is geographically limited in LMICs and underscore the need for the development of PROMs that are explicitly validated for LMIC populations.

There are several possible explanations for the limited use of PROMs in LMICs. First, the use of PROMs in breast surgery is contingent on the access to and delivery of immediate breast reconstruction. In LMICs, factors which may limit the availability and accessibility of breast reconstruction include high financial costs and disproportionate number of specialty-trained surgeons relative to the need [48, 55–59]. Moreover, while legislation mandates insurance coverage for breast reconstruction in HICs like the United States [60], many LMICs may classify breast reconstruction as a cosmetic procedure, requiring out-of-pocket payment [55]. This further increases costs and reduces affordable access. Second, the use of PROMs often requires additional staffing, and technological and data resources [61–63]. This may cause undue strain on healthcare delivery in certain LMIC contexts. Third, studies have shown that many PROMs exceed recommended readability and literacy standards [64–66], which may exacerbate adoption in certain LMICs that have populations with lower education and literacy levels. Furthermore, the availability of translated versions of PROMs is limited, thereby restricting their use among non-English speaking populations in LMICs. In addition, certain PROMs may be deemed culturally inappropriate or irrelevant [67]. For example, one study in our review found that the BREAST-Q may not be optimal for Chinese women who focus on breast shape when clothed [32].

This review highlights that the administration of PROMs after breast reconstruction is geographically limited in LMICs. Most (84.8%) of the studies were conducted in upper middle-income countries, with 15.2% of studies in lower middle-income countries and no studies in low-income countries. While this review included 33 studies, only 10 different countries were represented, with multiple studies conducted in China, Brazil, Mexico, Turkey, and Egypt. The large majority (84.8%) of studies were completed in urban settings, primarily in academic medical centers. A scoping review conducted by Masyuko et al. on the use of PROMs among patients with diabetes and hypertension noted similar findings; of the 68 included studies, 57% were conducted in upper-middle-income countries and 6% in low-income countries, although information on urban versus rural settings was not included [68]. In the present study, none of the studies were conducted in low-income countries, likely due to limited access to breast reconstruction in rural areas or non-academic medical centers [69]. Together, these findings elucidate not only that PROM use is unevenly represented among LMICs, but also that within LMICs, PROM use is especially limited among low-income countries and in rural settings.

While most studies incorporated the use of breast- and/or breast reconstruction-specific PROMs, only 45.5% of studies included a PROM that had been explicitly validated for their populations of interest. Translation and adaption of PROMs to a different language and culture often involve a rigorous, multistep process [70] that requires resources that may be limited in LMICs. The development and validation of PROMs that are inclusive and representative of diverse populations in HICs will expand the appropriate usage of PROMs in LMICs. The importance of language and cross-cultural validation of PROMs has been cited previously in other contexts [71–74] and our current study reiterates this finding in LMICs.

Our study is not without limitations. Only studies written in English were included. Given the focus of this review on LMICs, this may have resulted in the exclusion of several otherwise relevant studies. Studies conducted in LMICs may not have been published in indexed journals. In addition, studies included did not consistently report details on the type of breast reconstruction performed, method and setting of PROM administration, PROM validation, or the response rate of PROMs. Therefore, these variables could not be comprehensively analyzed. Finally, many studies did not include potential barriers and facilitators of PROM use, limiting our understanding of the challenges that need to be considered when administering PROMs in LMICs.

Although this scoping review focused on breast reconstruction, it underscores that PROM use overall may be limited in LMICs. The administration and routine clinical implementation of PROMs are challenging even in HICs due to barriers including interference with clinical workflows, technical difficulties, and low patient response rates [75]. To address these barriers, support strategies targeting pre-implementation, implementation, and post-implementation stages have been used based on context-specific enabling factors [76]. In LMICs, such barriers are compounded by inadequate resources, lack of education on PROMs, and limited availability of translated versions. Although this review examined PROM use in LMICs, it is notable that none of the studies in this review were conducted in low-income countries. As such, the implementation of appropriate interventions should be guided by the barriers and facilitators within the geographical area of interest to address the challenges of PROM use and guide effective PROM development and administration globally. We suggest several recommendations. To increase the utilization of PROMs in LMICs, future efforts should involve incorporating education (e.g., training of surgeons in LMICs) related to PROMs into global surgery efforts. In addition, given that LMICs have limited healthcare resources, the process of PROM development in HICs should ensure easy adaptability to the different languages and cultural contexts of LMICs. Moreover, studies of PROM administration in HICs should be clear and transparent in reporting barriers and facilitators to PROM use (e.g., costs, staffing and technological requirements) to appropriately set expectations for implementation in LMICs and to allow for further improvements in the development and implementation of PROMs.

## Conclusion

Despite the burden of breast cancer in LMICs and the importance of utilization of PROMs in measuring HRQL among breast cancer patients after breast reconstruction, administration of PROMs in LMICs is limited. Further research is necessary to understand the impact of breast reconstruction on HRQL as well as barriers and facilitators of PROM implementation in LMICs. Addressing challenges of PROM administration in LMICs, including effective utilization of limited resources as well as translation and adaptation of PROMs based on sociocultural contexts, will be imperative to promote equitable care of breast reconstruction patients globally.

### Electronic supplementary material

Below is the link to the electronic supplementary material.

Supplemental Digital Content 1: Search terms and strategy.

## Data Availability

The datasets used during the current study are available from the corresponding author on reasonable request.
