# Supplementary material for: Use of patient-reported outcome measures after breast reconstruction in low- and middle-income countries: a scoping review
Source: J Patient Rep Outcomes. 2024 Feb 28;8:25. doi: 10.1186/s41687-024-00687-y (PMC10899941; doi:10.1186/s41687-024-00687-y)
Supplement: Supplementary file 1 — Supplemental Digital Content 1: Search terms and strategy. [file 41687_2024_687_MOESM1_ESM.docx]

**Supplemental Digital Content 1.** Search terms and strategy

MEDLINE (Ovid)

Ovid MEDLINE(R) and Epub Ahead of Print, In-Process, In-Data-Review & Other Non-Indexed Citations, Daily and Versions 1946 to August 26, 2022

August 28, 2022

381 Records

1. exp patient reported outcome measures/ or exp patient satisfaction/ or exp quality of life/ or (((patient* or self) adj1 (report* or rate or rating or rated)) and ((report* or rate or rating or rated) adj3 (outcome* or measur* or symptom*))).ab,kf,kw,ti or ((patientreport* or selfreport*) adj3 (outcome* OR measur* OR symptom*)).ab,kf,kw,ti or (prom or proms or promis or eortc or qlq-c30 or qlqc30 or qlq-br23 or qlqbr23 or qlq-lmc21 or qlqlmc21 or breast-q or fact-es or factes or short form 36 or fact-b or factb or satisfaction or quality of life or sexual* or well being or wellbeing).ab,kf,kw,ti or ((health or health related or physical) adj3 function*).ab,kf,kw,ti 1054598

2. exp breast neoplasms/su or exp mammoplasty/ or exp mastectomy/ or exp breast implantation/ or exp breast implants/ or (reconstruct* or mammoplast* or mammaplast* or mastectomy or lumpectom* or implant* or deep inferior epigastric perforator or diep or deep inferior epigastric artery perforator or dieap or transverse rectus abdominis muscle or tram or anterior lateral thigh or gluteal artery perforator or latissimus dorsi or transverse upper gracilis or flap or flaps or surger*).ab,kf,kw,ti 2132478

3. exp breast neoplasms/ or (breast* adj3 (cancer* or neoplas* or carcinoma* or adenocarcinoma*)).ab,kf,kw,ti 443122

4. exp developing countries or ((developing or less developed or third world or under developed or middle income or low income or underserved or under served or deprived or poor*) adj1 (countr* or nation* or state*)).ab,kf,kw,ti or (resource* adj2 (poor or limiting or limited or low or constrain*)).ab,kf,kw,ti or exp africa/ or exp asia/ or exp south America/ or exp latin America/ or exp central america/ or (lmic or lmics or africa or asia or south america* or latin america* or central america* or afghanistan* or albania* or algeria* or samoa* or angola* or argentina* or armenia* or azerbaijan* or bangladesh* or belarus* or belize* or benin* or bhutan* or bolivia* or bosnia* or herzegovina* or botswana* or brazil* or bulgaria* or burkin* or burundi* or cabo verde* or cape verde* or cambodia* or cameroon* or central africa* or chad* or china or chinese or colombia* or comoros or comorian* or congo* or costa rica* or cote d ivoire or ivorian* or ivory coast or cuba* or djibouti* or dominica* or ecuador* or egypt* or el salvador or salvadoran* or guinea* or eritrea* or eswatini* or swaziland* or ethiopia* or fiji* or gabon* or gambia* or "republic of georgia" or ghana* or grenada* or guatemala* or guyana* or haiti* or honduras* or india* or indonesia* or iran* or iraq* or jamaica* or jordan* or kazakhstan* or kenya* or kiribati* or "democratic people s republic of korea" or north korea* or kosovo or kosovar* or kyrgyz* or lao or laos or laotian* or lebanon* or lesotho or liberia* or libya* or madagascar* or malawi* or malaysia* or maldives* or mali or malian or malians or marshall island* or mauritania* or mexico or mexican* or micronesia* or moldova* or mongolia* or montenegr* or morocc* or mozambi* or myanmar* or namibia* or nepal* or nicaragua* or niger or nigerien* or nigeria* or macedonia* or palau* or pakistan* or paragua* or peru* or philippines or filipino* or russia* or rwanda* or sao tome and principe or senegal* or serbia* or sierra leone* or solomon island* or somalia* or south africa* or sudan* or sri lank* or surinam* or st lucia* or saint lucia* or st vincent* or saint vincent* or grenadines or syria* or tajikistan* or tanzania* or thailand or thai or thais or timor* or togo or togolese* or tonga* or tunisia* or turkey or turks or turkish or turkmenistan* or tuvalu* or uganda* or ukrain* or uzbekistan* or vanuatu* or venezuela* or vietnam* or viet nam* or west bank or gaza or gazan* or yemen* or zambia* or zimbabwe*).ab,kf,kw,ti 1725739

5. 1 and 2 and 3 and 4 381

Embase (Elsevier, 1974-)

August 28, 2022

445 Records

1. 'patient-reported outcome'/de OR 'patient satisfaction'/exp OR 'quality of life'/exp OR ((patientreport* OR selfreport*) NEAR/3 (outcome* OR measur* OR symptom*)):ab,kw,ti OR ((health OR 'health related' OR physical) NEAR/3 function*):ab,kw,ti OR (prom OR proms OR promis OR eortc OR 'qlq-c30' OR qlqc30 OR 'qlq-br23' OR qlqbr23 OR 'qlq-lmc21' OR qlqlmc21 OR 'breast-q' OR 'fact-es' OR factes OR 'short form 36' OR 'fact-b' OR factb OR satisfaction OR 'quality of life' OR sexual* OR 'well being' OR wellbeing):ab,kw,ti OR (((patient* OR self) NEAR/1 (report* OR rate OR rating OR rated)):ab,kw,ti AND ((report* OR rate OR rating OR rated) NEAR/3 (outcome* OR measur* OR symptom*)):ab,kw,ti) 1228049

2. 'breast reconstruction'/de OR 'mastectomy'/exp OR 'breast endoprosthesis'/exp OR (reconstruct* OR mammoplast* OR mammaplast* OR mastectomy OR lumpectom* OR implant* OR 'deep inferior epigastric perforator' OR diep OR 'deep inferior epigastric artery perforator' OR dieap OR 'transverse rectus abdominis muscle' OR tram OR 'anterior lateral thigh' OR 'gluteal artery perforator' OR 'latissimus dorsi' OR 'transverse upper gracilis' OR flap OR flaps OR surger*):ab,kw,ti 2323992

3. 'breast cancer'/exp OR (breast* NEAR/3 (cancer* or neoplas* or carcinoma* or adenocarcinoma*)):ab,kw,ti 594369

4. 'developing country'/exp OR 'low income country'/de OR 'middle income country'/de OR ((developing OR 'less developed' OR 'third world' OR 'under developed' OR 'middle income' OR 'low income' OR underserved OR 'under served' OR deprived OR poor*) NEAR/1 (countr* OR nation* OR state* OR population*)):ab,kw,ti OR 'Africa'/de OR 'Africa south of the Sahara'/de OR 'North Africa'/de OR 'Asia'/de OR 'Far East'/de OR 'Middle East'/de OR 'South Asia'/de OR 'South and Central America'/de OR 'South America'/de OR 'Central America'/de OR 'Caribbean'/de OR (lmic OR lmics OR africa OR asia OR 'south america*' OR 'latin america*' OR 'central america*' OR afghanistan* OR albania* OR algeria* OR samoa* OR angola* OR argentina* OR armenia* OR azerbaijan* OR bangladesh* OR belarus* OR belize* OR benin* OR bhutan* OR bolivia* OR bosnia* OR herzegovina* OR botswana* OR brazil* OR bulgaria* OR burkin* OR burundi* OR 'cabo verde*' OR 'cape verde*' OR cambodia* OR cameroon* OR 'central africa*' OR chad* OR china OR chinese OR colombia* OR comoros OR comorian* OR congo* OR 'costa rica*' OR 'cote d ivoire*' OR ivorian* OR 'ivory coast' OR cuba* OR djibouti* OR dominica* OR ecuador* OR egypt* OR 'el salvador' OR salvadoran* OR guinea* OR eritrea* OR eswatini* OR swaziland* OR ethiopia* OR fiji* OR gabon* OR gambia* OR 'republic of georgia' OR ghana* OR grenada* OR guatemala* OR guyana* OR haiti* OR honduras* OR india* OR indonesia* OR iran* OR iraq* OR jamaica* OR jordan* OR kazakhstan* OR kenya* OR kiribati* OR 'democratic people s republic of korea' OR 'north korea*' OR kosovo OR kosovar* OR kyrgyz* OR lao OR laos OR laotian* OR lebanon* OR lesotho OR liberia* OR libya* OR madagascar* OR malawi* OR malaysia* OR maldives* OR mali OR malian OR malians OR 'marshall island*' OR mauritania* OR mexico OR mexican* OR micronesia* OR moldova* OR mongolia* OR montenegr* OR morocc* OR mozambi* OR myanmar* OR namibia* OR nepal* OR nicaragua* OR niger OR nigerien* OR nigeria* OR macedonia* OR palau* OR pakistan* OR paragua* OR peru* OR philippines OR filipino* OR russia* OR rwanda* OR 'sao tome and principe' OR senegal* OR serbia* OR 'sierra leone*' OR 'solomon island*' OR somalia* OR 'south africa*' OR sudan* OR 'sri lank*' OR surinam* OR 'st lucia*' OR 'saint lucia*' OR 'st vincent*' OR 'saint vincent*' OR grenadines OR syria* OR tajikistan* OR tanzania* OR thailand OR thai OR thais OR timor* OR togo OR togolese* OR tonga* OR tunisia* OR turkey OR turks OR turkish OR turkmenistan* OR tuvalu* OR uganda* OR ukrain* OR uzbekistan* OR vanuatu* OR venezuela* OR vietnam* OR 'viet nam*' OR 'west bank' OR gaza OR gazan* OR yemen* OR zambia* OR zimbabwe*):ab,kw,ti 1931404

5. #1 AND #2 AND #3 AND #4 747

6. #5 NOT 'conference abstract'/it 445

Web of Science Core Collection (Clarivate)

Editions = A&HCI , BKCI-SSH , BKCI-S , CCR-EXPANDED , ESCI , IC , CPCI-SSH , CPCI-S , SCI-EXPANDED , SSCI

August 28, 2022

557 Records

1. TS=(((patientreport* OR selfreport*) NEAR/3 (outcome* OR measur* OR symptom*)) OR ((health OR "health related" OR physical) NEAR/3 function*) OR prom OR proms OR promis OR eortc OR "qlq-c30" OR qlqc30 OR "qlq-br23" OR qlqbr23 OR "qlq-lmc21" OR qlqlmc21 OR "breast-q" OR "fact-es" OR factes OR "short form 36" OR "fact-b" OR factb OR satisfaction OR "quality of life" OR sexual* OR "well being" OR wellbeing OR (((patient* OR self) NEAR/1 (report* OR rate OR rating OR rated)) AND ((report* OR rate OR rating OR rated) NEAR/3 (outcome* OR measur* OR symptom*)))) 1504845

2. TS=(reconstruct* OR mammoplast* OR mammaplast* OR mastectomy OR lumpectom* OR implant* OR "deep inferior epigastric perforator" OR diep OR "deep inferior epigastric artery perforator" OR dieap OR "transverse rectus abdominis muscle" OR tram OR "anterior lateral thigh" OR "gluteal artery perforator" OR "latissimus dorsi" OR "transverse upper gracilis" OR flap OR flaps OR surger*) 2604012

3. TS=(breast* NEAR/3 (cancer* or neoplas* or carcinoma* or adenocarcinoma*)) 617298

4. TS=(((developing OR "less developed" OR "third world" OR "under developed" OR "middle income" OR "low income" OR underserved OR "under served" OR deprived OR poor*) NEAR/1 (countr* OR nation* OR state* OR population* OR area*)) OR (resource* NEAR/2 (poor OR limiting OR limited OR low OR constrain*)) OR lmic OR lmics OR africa OR asia OR "south america*" OR "latin america*" OR "central america*" OR afghanistan* OR albania* OR algeria* OR samoa* OR angola* OR argentina* OR armenia* OR azerbaijan* OR bangladesh* OR belarus* OR belize* OR benin* OR bhutan* OR bolivia* OR bosnia* OR herzegovina* OR botswana* OR brazil* OR bulgaria* OR burkin* OR burundi* OR "cabo verde*" OR "cape verde*" OR cambodia* OR cameroon* OR "central africa*" OR chad* OR china OR chinese OR colombia* OR comoros OR comorian* OR congo* OR costa rica* OR "cote d ivoire*" OR ivorian* OR "ivory coast" OR cuba* OR djibouti* OR dominica* OR ecuador* OR egypt* OR "el salvador" OR salvadoran* OR guinea* OR eritrea* OR eswatini* OR swaziland* OR ethiopia* OR fiji* OR gabon* OR gambia* OR "republic of georgia" OR ghana* OR grenada* OR guatemala* OR guyana* OR haiti* OR honduras* OR india* OR indonesia* OR iran* OR iraq* OR jamaica* OR jordan* OR kazakhstan* OR kenya* OR kiribati* OR "democratic people s republic of korea" OR "north korea*" OR kosovo OR kosovar* OR kyrgyz* OR lao OR laos OR laotian* OR lebanon* OR lesotho OR liberia* OR libya* OR madagascar* OR malawi* OR malaysia* OR maldives* OR mali OR malian OR malians OR "marshall island*" OR mauritania* OR mexico OR mexican* OR micronesia* OR moldova* OR mongolia* OR montenegr* OR morocc* OR mozambi* OR myanmar* OR namibia* OR nepal* OR nicaragua* OR niger OR nigerien* OR nigeria* OR macedonia* OR pakistan* OR paragua* OR peru* OR philippines OR filipino* OR russia* OR rwanda* OR "sao tome*" OR senegal* OR serbia* OR "sierra leone*" OR "solomon island*" OR somalia* OR "south africa*" OR sudan* OR "sri lank*" OR "st lucia*" OR "saint lucia*" OR "st vincent*" OR "saint vincent*" OR grenadines OR surinam* OR syria* OR tajikistan* OR tanzania* OR thailand OR thai OR thais OR timor* OR togo OR togolese* OR tonga* OR tunisia* OR turkey OR turks OR turkish OR turkmenistan* OR tuvalu* OR uganda* OR ukrain* OR uzbekistan* OR vanuatu* OR venezuela* OR vietnam* OR "viet nam*" OR "west bank" OR gaza OR gazan* OR yemen* OR zambia* OR zimbabwe*) 5386628

5. #1 AND #2 AND #3 AND #4 557

CINAHL Complete (EBSCO)

August 28, 2022

155 Records

1, MH ("Patient-Reported Outcomes+" OR "Patient Satisfaction" OR "Quality of Life") OR TI (((patientreport* OR selfreport*) N3 (outcome* OR measur* OR symptom*)) OR ((health OR "health related" OR physical) N3 function*) OR prom OR proms OR promis OR eortc OR "qlq-c30" OR qlqc30 OR "qlq-br23" OR qlqbr23 OR "qlq-lmc21" OR qlqlmc21 OR "breast-q" OR "fact-es" OR factes OR "short form 36" OR "fact-b" OR factb OR satisfaction OR "quality of life" OR sexual* OR "well being" OR wellbeing OR (((patient* OR self) N1 (report* OR rate OR rating OR rated)) AND ((report* OR rate OR rating OR rated) N3 (outcome* OR measur* OR symptom*)))) OR AB (((patientreport* OR selfreport*) N3 (outcome* OR measur* OR symptom*)) OR ((health OR "health related" OR physical) N3 function*) OR prom OR proms OR promis OR eortc OR "qlq-c30" OR qlqc30 OR "qlq-br23" OR qlqbr23 OR "qlq-lmc21" OR qlqlmc21 OR "breast-q" OR "fact-es" OR factes OR "short form 36" OR "fact-b" OR factb OR satisfaction OR "quality of life" OR sexual* OR "well being" OR wellbeing OR (((patient* OR self) N1 (report* OR rate OR rating OR rated)) AND ((report* OR rate OR rating OR rated) N3 (outcome* OR measur* OR symptom*)))) 510921

2. MH ("Breast Reconstruction" OR " Mastectomy+") OR TI (reconstruct* OR mammoplast* OR mammaplast* OR mastectomy OR lumpectom* OR implant* OR "deep inferior epigastric perforator" OR diep OR "deep inferior epigastric artery perforator" OR dieap OR "transverse rectus abdominis muscle" OR tram OR "anterior lateral thigh" OR "gluteal artery perforator" OR "latissimus dorsi" OR "transverse upper gracilis" OR flap OR flaps OR surger*) OR AB (reconstruct* OR mammoplast* OR mammaplast* OR mastectomy OR lumpectom* OR implant* OR "deep inferior epigastric perforator" OR diep OR "deep inferior epigastric artery perforator" OR dieap OR "transverse rectus abdominis muscle" OR tram OR "anterior lateral thigh" OR "gluteal artery perforator" OR "latissimus dorsi" OR "transverse upper gracilis" OR flap OR flaps OR surger*) 423882

3. MH ("Breast Neoplasms") OR TI (breast* N3 (cancer* or neoplas* or carcinoma* or adenocarcinoma*)) OR AB (breast* N3 (cancer* or neoplas* or carcinoma* or adenocarcinoma*)) 117052

4. MH ("Developing Countries" OR "Low and Middle Income Countries") OR TI (((developing OR "less developed" OR "third world" OR "under developed" OR "middle income" OR "low income" OR underserved OR "under served" OR deprived OR poor*) N1 (countr* OR nation* OR state* OR population* OR)) OR lmic OR lmics OR africa OR asia OR "south america*" OR "latin america*" OR "central america*" OR afghanistan* OR albania* OR algeria* OR samoa* OR angola* OR argentina* OR armenia* OR azerbaijan* OR bangladesh* OR belarus* OR belize* OR benin* OR bhutan* OR bolivia* OR bosnia* OR herzegovina* OR botswana* OR brazil* OR bulgaria* OR burkin* OR burundi* OR "cabo verde*" OR "cape verde*" OR cambodia* OR cameroon* OR "central africa*" OR chad* OR china OR chinese OR colombia* OR comoros OR comorian* OR congo* OR costa rica* OR "cote d ivoire*" OR ivorian* OR "ivory coast" OR cuba* OR djibouti* OR dominica* OR ecuador* OR egypt* OR "el salvador" OR salvadoran* OR guinea* OR eritrea* OR eswatini* OR swaziland* OR ethiopia* OR fiji* OR gabon* OR gambia* OR "republic of georgia" OR ghana* OR grenada* OR guatemala* OR guyana* OR haiti* OR honduras* OR india* OR indonesia* OR iran* OR iraq* OR jamaica* OR jordan* OR kazakhstan* OR kenya* OR kiribati* OR "democratic people s republic of korea" OR "north korea*" OR kosovo OR kosovar* OR kyrgyz* OR lao OR laos OR laotian* OR lebanon* OR lesotho OR liberia* OR libya* OR madagascar* OR malawi* OR malaysia* OR maldives* OR mali OR malian OR malians OR "marshall island*" OR mauritania* OR mexico OR mexican* OR micronesia* OR moldova* OR mongolia* OR montenegr* OR morocc* OR mozambi* OR myanmar* OR namibia* OR nepal* OR nicaragua* OR niger OR nigerien* OR nigeria* OR macedonia* OR pakistan* OR paragua* OR peru* OR philippines OR filipino* OR russia* OR rwanda* OR "sao tome*" OR senegal* OR serbia* OR "sierra leone*" OR "solomon island*" OR somalia* OR "south africa*" OR sudan* OR "sri lank*" OR "st lucia*" OR "saint lucia*" OR "st vincent*" OR "saint vincent*" OR grenadines OR surinam* OR syria* OR tajikistan* OR tanzania* OR thailand OR thai OR thais OR timor* OR togo OR togolese* OR tonga* OR tunisia* OR turkey OR turks OR turkish OR turkmenistan* OR tuvalu* OR uganda* OR ukrain* OR uzbekistan* OR vanuatu* OR venezuela* OR vietnam* OR "viet nam*" OR "west bank" OR gaza OR gazan* OR yemen* OR zambia* OR zimbabwe*) OR AB (((developing OR "less developed" OR "third world" OR "under developed" OR "middle income" OR "low income" OR underserved OR "under served" OR deprived OR poor*) N1 (countr* OR nation* OR state* OR population* OR area*)) OR (resource* N2 (poor OR limiting OR limited OR low OR constrain*)) OR lmic OR lmics OR africa OR asia OR "south america*" OR "latin america*" OR "central america*" OR afghanistan* OR albania* OR algeria* OR samoa* OR angola* OR argentina* OR armenia* OR azerbaijan* OR bangladesh* OR belarus* OR belize* OR benin* OR bhutan* OR bolivia* OR bosnia* OR herzegovina* OR botswana* OR brazil* OR bulgaria* OR burkin* OR burundi* OR "cabo verde*" OR "cape verde*" OR cambodia* OR cameroon* OR "central africa*" OR chad* OR china OR chinese OR colombia* OR comoros OR comorian* OR congo* OR costa rica* OR "cote d ivoire*" OR ivorian* OR "ivory coast" OR cuba* OR djibouti* OR dominica* OR ecuador* OR egypt* OR "el salvador" OR salvadoran* OR guinea* OR eritrea* OR eswatini* OR swaziland* OR ethiopia* OR fiji* OR gabon* OR gambia* OR "republic of georgia" OR ghana* OR grenada* OR guatemala* OR guyana* OR haiti* OR honduras* OR india* OR indonesia* OR iran* OR iraq* OR jamaica* OR jordan* OR kazakhstan* OR kenya* OR kiribati* OR "democratic people s republic of korea" OR "north korea*" OR kosovo OR kosovar* OR kyrgyz* OR lao OR laos OR laotian* OR lebanon* OR lesotho OR liberia* OR libya* OR madagascar* OR malawi* OR malaysia* OR maldives* OR mali OR malian OR malians OR "marshall island*" OR mauritania* OR mexico OR mexican* OR micronesia* OR moldova* OR mongolia* OR montenegr* OR morocc* OR mozambi* OR myanmar* OR namibia* OR nepal* OR nicaragua* OR niger OR nigerien* OR nigeria* OR macedonia* OR pakistan* OR paragua* OR peru* OR philippines OR filipino* OR russia* OR rwanda* OR "sao tome*" OR senegal* OR serbia* OR "sierra leone*" OR "solomon island*" OR somalia* OR "south africa*" OR sudan* OR "sri lank*" OR "st lucia*" OR "saint lucia*" OR "st vincent*" OR "saint vincent*" OR grenadines OR surinam* OR syria* OR tajikistan* OR tanzania* OR thailand OR thai OR thais OR timor* OR togo OR togolese* OR tonga* OR tunisia* OR turkey OR turks OR turkish OR turkmenistan* OR tuvalu* OR uganda* OR ukrain* OR uzbekistan* OR vanuatu* OR venezuela* OR vietnam* OR "viet nam*" OR "west bank" OR gaza OR gazan* OR yemen* OR zambia* OR zimbabwe*) 488236

5. S1 AND S2 AND S3 AND S4 155

PsycINFO (EBSCO)

20220828

56 Records

1. DE "Patient Reported Outcome Measures" OR "Sexual Satisfaction" OR "Marital Satisfaction" OR "Quality of Life" OR "Health Related Quality of Life") OR TI (((patientreport* OR selfreport*) N3 (outcome* OR measur* OR symptom*)) OR ((health OR "health related" OR physical) N3 function*) OR prom OR proms OR promis OR eortc OR "qlq-c30" OR qlqc30 OR "qlq-br23" OR qlqbr23 OR "qlq-lmc21" OR qlqlmc21 OR "breast-q" OR "fact-es" OR factes OR "short form 36" OR "fact-b" OR factb OR satisfaction OR "quality of life" OR sexual* OR "well being" OR wellbeing OR (((patient* OR self) N1 (report* OR rate OR rating OR rated)) AND ((report* OR rate OR rating OR rated) N3 (outcome* OR measur* OR symptom*)))) OR AB (((patientreport* OR selfreport*) N3 (outcome* OR measur* OR symptom*)) OR ((health OR "health related" OR physical) N3 function*) OR prom OR proms OR promis OR eortc OR "qlq-c30" OR qlqc30 OR "qlq-br23" OR qlqbr23 OR "qlq-lmc21" OR qlqlmc21 OR "breast-q" OR "fact-es" OR factes OR "short form 36" OR "fact-b" OR factb OR satisfaction OR "quality of life" OR sexual* OR "well being" OR wellbeing OR (((patient* OR self) N1 (report* OR rate OR rating OR rated)) AND ((report* OR rate OR rating OR rated) N3 (outcome* OR measur* OR symptom*)))) 529860

2. DE ("Mastectomy") OR TI (reconstruct* OR mammoplast* OR mammaplast* OR mastectomy OR lumpectom* OR implant* OR "deep inferior epigastric perforator" OR diep OR "deep inferior epigastric artery perforator" OR dieap OR "transverse rectus abdominis muscle" OR tram OR "anterior lateral thigh" OR "gluteal artery perforator" OR "latissimus dorsi" OR "transverse upper gracilis" OR flap OR flaps OR surger*) OR AB (reconstruct* OR mammoplast* OR mammaplast* OR mastectomy OR lumpectom* OR implant* OR "deep inferior epigastric perforator" OR diep OR "deep inferior epigastric artery perforator" OR dieap OR "transverse rectus abdominis muscle" OR tram OR "anterior lateral thigh" OR "gluteal artery perforator" OR "latissimus dorsi" OR "transverse upper gracilis" OR flap OR flaps OR surger*) 67653

3. DE ("Breast Neoplasms") OR TI (breast* N3 (cancer* or neoplas* or carcinoma* or adenocarcinoma*)) OR AB (breast* N3 (cancer* or neoplas* or carcinoma* or adenocarcinoma*)) 16001

4. DE ("Developing Countries") OR TI (((developing OR "less developed" OR "third world" OR "under developed" OR "middle income" OR "low income" OR underserved OR "under served" OR deprived OR poor*) N1 (countr* OR nation* OR state* OR population* OR)) OR lmic OR lmics OR africa OR asia OR "south america*" OR "latin america*" OR "central america*" OR afghanistan* OR albania* OR algeria* OR samoa* OR angola* OR argentina* OR armenia* OR azerbaijan* OR bangladesh* OR belarus* OR belize* OR benin* OR bhutan* OR bolivia* OR bosnia* OR herzegovina* OR botswana* OR brazil* OR bulgaria* OR burkin* OR burundi* OR "cabo verde*" OR "cape verde*" OR cambodia* OR cameroon* OR "central africa*" OR chad* OR china OR chinese OR colombia* OR comoros OR comorian* OR congo* OR costa rica* OR "cote d ivoire*" OR ivorian* OR "ivory coast" OR cuba* OR djibouti* OR dominica* OR ecuador* OR egypt* OR "el salvador" OR salvadoran* OR guinea* OR eritrea* OR eswatini* OR swaziland* OR ethiopia* OR fiji* OR gabon* OR gambia* OR "republic of georgia" OR ghana* OR grenada* OR guatemala* OR guyana* OR haiti* OR honduras* OR india* OR indonesia* OR iran* OR iraq* OR jamaica* OR jordan* OR kazakhstan* OR kenya* OR kiribati* OR "democratic people s republic of korea" OR "north korea*" OR kosovo OR kosovar* OR kyrgyz* OR lao OR laos OR laotian* OR lebanon* OR lesotho OR liberia* OR libya* OR madagascar* OR malawi* OR malaysia* OR maldives* OR mali OR malian OR malians OR "marshall island*" OR mauritania* OR mexico OR mexican* OR micronesia* OR moldova* OR mongolia* OR montenegr* OR morocc* OR mozambi* OR myanmar* OR namibia* OR nepal* OR nicaragua* OR niger OR nigerien* OR nigeria* OR macedonia* OR pakistan* OR paragua* OR peru* OR philippines OR filipino* OR russia* OR rwanda* OR "sao tome*" OR senegal* OR serbia* OR "sierra leone*" OR "solomon island*" OR somalia* OR "south africa*" OR sudan* OR "sri lank*" OR "st lucia*" OR "saint lucia*" OR "st vincent*" OR "saint vincent*" OR grenadines OR surinam* OR syria* OR tajikistan* OR tanzania* OR thailand OR thai OR thais OR timor* OR togo OR togolese* OR tonga* OR tunisia* OR turkey OR turks OR turkish OR turkmenistan* OR tuvalu* OR uganda* OR ukrain* OR uzbekistan* OR vanuatu* OR venezuela* OR vietnam* OR "viet nam*" OR "west bank" OR gaza OR gazan* OR yemen* OR zambia* OR zimbabwe*) OR AB (((developing OR "less developed" OR "third world" OR "under developed" OR "middle income" OR "low income" OR underserved OR "under served" OR deprived OR poor*) N1 (countr* OR nation* OR state* OR population* OR area*)) OR (resource* N2 (poor OR limiting OR limited OR low OR constrain*)) OR lmic OR lmics OR africa OR asia OR "south america*" OR "latin america*" OR "central america*" OR afghanistan* OR albania* OR algeria* OR samoa* OR angola* OR argentina* OR armenia* OR azerbaijan* OR bangladesh* OR belarus* OR belize* OR benin* OR bhutan* OR bolivia* OR bosnia* OR herzegovina* OR botswana* OR brazil* OR bulgaria* OR burkin* OR burundi* OR "cabo verde*" OR "cape verde*" OR cambodia* OR cameroon* OR "central africa*" OR chad* OR china OR chinese OR colombia* OR comoros OR comorian* OR congo* OR costa rica* OR "cote d ivoire*" OR ivorian* OR "ivory coast" OR cuba* OR djibouti* OR dominica* OR ecuador* OR egypt* OR "el salvador" OR salvadoran* OR guinea* OR eritrea* OR eswatini* OR swaziland* OR ethiopia* OR fiji* OR gabon* OR gambia* OR "republic of georgia" OR ghana* OR grenada* OR guatemala* OR guyana* OR haiti* OR honduras* OR india* OR indonesia* OR iran* OR iraq* OR jamaica* OR jordan* OR kazakhstan* OR kenya* OR kiribati* OR "democratic people s republic of korea" OR "north korea*" OR kosovo OR kosovar* OR kyrgyz* OR lao OR laos OR laotian* OR lebanon* OR lesotho OR liberia* OR libya* OR madagascar* OR malawi* OR malaysia* OR maldives* OR mali OR malian OR malians OR "marshall island*" OR mauritania* OR mexico OR mexican* OR micronesia* OR moldova* OR mongolia* OR montenegr* OR morocc* OR mozambi* OR myanmar* OR namibia* OR nepal* OR nicaragua* OR niger OR nigerien* OR nigeria* OR macedonia* OR pakistan* OR paragua* OR peru* OR philippines OR filipino* OR russia* OR rwanda* OR "sao tome*" OR senegal* OR serbia* OR "sierra leone*" OR "solomon island*" OR somalia* OR "south africa*" OR sudan* OR "sri lank*" OR "st lucia*" OR "saint lucia*" OR "st vincent*" OR "saint vincent*" OR grenadines OR surinam* OR syria* OR tajikistan* OR tanzania* OR thailand OR thai OR thais OR timor* OR togo OR togolese* OR tonga* OR tunisia* OR turkey OR turks OR turkish OR turkmenistan* OR tuvalu* OR uganda* OR ukrain* OR uzbekistan* OR vanuatu* OR venezuela* OR vietnam* OR "viet nam*" OR "west bank" OR gaza OR gazan* OR yemen* OR zambia* OR zimbabwe*) 358670

5. S1 AND S2 AND S3 AND S4 56
